# Microstructure, Mechanical Properties, and Corrosion Behavior in Al-5.6Zn-2.5Mg-1.6Cu-0.2Cr Alloy with Minor Yttrium Addition

**DOI:** 10.3390/ma18040875

**Published:** 2025-02-17

**Authors:** Ting Yao, Daihong Xiao, Yingjie Yan, Wensheng Liu

**Affiliations:** 1 Shaanxi Nonferrous Yulin New Material Group Co., Ltd., Yulin 719099, China; dinglangkuangdang@163.com; 2 Powder Metallurgy Research Institute, Central South University, Changsha 410083, China; yyyblossom@163.com (Y.Y.); liuwensheng@csu.edu.cn (W.L.); 3 China Nonferrous Metals (Tianjin) New Material Technology Co., Ltd., Tianjing 300380, China

**Keywords:** Al-Zn-Mg-Cu-Cr alloy, yttrium addition, microstructure, mechanical properties, corrosion behavior

## Abstract

This study systematically investigated the effects of the addition of the rare earth element yttrium (Y) on the microstructural evolution, mechanical properties, and corrosion behavior of as-extruded Al-5.6Zn-2.5Mg-1.6Cu-0.20Cr (wt.%) alloy through comprehensive characterization techniques, including X-ray diffraction (XRD), tensile testing, corrosion analysis, and electron microscopy. Microstructural characterization demonstrated that the incorporation of yttrium resulted in significant refinement of secondary phase particles within the as-extruded alloy matrix. Moreover, quantitative analysis revealed a substantial increase in low-angle grain boundary (LAGB) density, dislocation density, and the formation of subgrain structures. Notably, the volume fraction of η′ strengthening precipitates showed a marked increase, accompanied by a corresponding reduction in the width of precipitate-free zones (PFZs) along grain boundaries. Following the T74 aging treatment, the alloy with 0.1 wt.% yttrium addition exhibited a remarkable improvement in intergranular corrosion resistance, with the maximum corrosion depth reduced to 107.8 μm. However, it should be noted that the exfoliation corrosion resistance exhibited an inverse correlation with increasing yttrium content, suggesting a concentration-dependent behavior in corrosion performance.

## 1. Introduction

Aluminum alloy systems, including Al-Cu-Mg, Al-Mg, Al-Mg-Si, and Al-Zn-Mg-Cu series, have garnered significant attention in multidisciplinary technological domains owing to their superior mechanical properties and structural adaptability. These advanced metallic materials demonstrate extensive applications across strategic industries such as aerospace engineering, defense technology, automotive manufacturing, and microelectronic packaging [1,2,3]. Specifically in the transportation sector, they serve as critical structural components in engine assemblies, piston-cylinder systems, and vehicular chassis architectures. Notably, Al-Zn-Mg-Cu alloys have emerged as premier candidates for load-bearing aerospace structures, particularly in aircraft wing spar fabrication, due to their exceptional combination of specific strength (σ/ρ), fracture toughness (K_IC_), and environmental degradation resistance. This material system exhibits substantial compositional complexity, characterized by multi-element synergistic interactions that significantly influence microstructural evolution during thermomechanical processing. The presence of transition metal constituents not only modulates recrystallization kinetics through Zener drag mechanisms but also governs the precipitation hardening behavior and intergranular corrosion susceptibility via η-phase (MgZn_2_) formation dynamics.

The 7075 aluminum alloy, recognized as the first generation of Al-Zn-Mg-Cu alloys, primarily consists of Al-(5.1–6.1) Zn, (2.1–2.9) Mg, (1.2–2.0) Cu, (0.18–0.28) Cr, (≤0.3) Mn, (≤0.5) Fe, and (≤0.4) Si (mass fraction) [3]. Due to its relatively low zinc content, the alloy is amenable to casting and exhibits favorable processing characteristics, allowing for various heat treatment options. Due to its high strength and toughness, the 7075 aluminum alloy continues to be widely used in aerospace applications to this day. However, the alloy contains elevated levels of iron and silicon impurities, with a total content reaching 0.9%, which adversely affects its overall properties, including strength, toughness, fatigue resistance, and corrosion resistance, compared to alloys 7050 and 7085. Consequently, researchers have focused on enhancing the comprehensive properties of 7075 aluminum alloy by regulating the total content and ratio of iron and silicon, as well as optimizing thermomechanical treatment processes [4,5,6].

The incorporation of yttrium (Y), a rare earth element characterized by its substantial atomic radius (0.18 nm), induces significant lattice strain (ε ≈ 1.8–2.3%) within the aluminum matrix, thereby enhancing solid solution strengthening through elastic stress field interactions [7]. From an economic perspective, yttrium serves as a cost-efficient surrogate for scandium (Sc), demonstrating comparable microstructural modification capabilities at lower material cost. In the context of 6063 aluminum alloy systems, yttrium addition (0.1 wt.%) facilitates phase transformation kinetics, converting detrimental β-AlFeSi plates into equiaxed α-AlFeSi precipitates, while simultaneously promoting the formation of complex intermetallic compounds including AlSiY, AlFeSi, and AlFeSiYMg phases [8]. This microstructural evolution effectively mitigates grain boundary segregation of Fe- and Si-rich phases, reducing their coverage fraction [8]. In hypereutectic Al-Si alloys, yttrium exerts a profound modification effect on silicon morphology, transforming coarse flake-like and acicular eutectic Si into refined fibrous structures with partial spheroidization [9]. Synergistic alloying strategies involving yttrium co-addition with other elements have shown remarkable microstructural control capabilities. Zhou et al. [10] demonstrated that combined Y-Si additions in 7056 aluminum alloys improve stress corrosion cracking (SCC) resistance, primarily through two mechanisms: (i) reduction of recrystallized fraction and (ii) increased sub-grain boundary density via formation of coherent (Al,Si)_3_(Zr,Y) dispersoids. Complementary research by Zhang et al. [11] revealed that Gd-Y co-addition in Al-Zn-Mg-Cu-Zr alloys effectively suppresses sub-grain coalescence during recovery processes, maintaining sub-grain size while completely inhibiting recrystallization through Zener pinning effects.

The existing literature reveals a limited number of research studies that concentrate on the application of rare earth element Y in 7075 aluminum alloy (Al-5.6Zn-2.5Mg-1.6Cu-0.20Cr). The purpose of this paper is to investigate the effects of the rare earth Y on the microstructure, mechanical properties, and corrosion behavior of the 7075 alloy, providing a reference for the alloying research of the alloy.

## 2. Materials and Method

The experimental materials utilized in this study consist of aluminum alloy, with the following composition: Al-5.6Zn-2.5Mg-1.6Cu-0.20Cr-0.1Fe-0.2Si (mass fraction, same as below). This alloy was modified with varying yttrium (Y) additions, specifically 0.0 Y (alloy 1), 0.1 Y (alloy 2), and 0.2 Y (alloy 3). The alloys were synthesized using high-purity raw materials, including electrolytic aluminum (99.99%), zinc (99.99%), and magnesium (99.97%), and master alloys such as Al-50% Cu, Al-20%Fe, Al-10%Si, Al-10%Cr, and Al-10 %Y (Xuzhou Fengrun Metal Materials Co., Ltd., Xuzhou, China), which were melted in a crucible. The molten alloy underwent argon degassing and refining processes prior to being cast into ingots. Following casting, the ingots were subjected to homogenization annealing at 470 °C for 24 h (Tianjin Zhonghua Furnace Corp., Tianjin, China). The homogenized ingots were then processed into round billets with a diameter of 98 mm, which were hot-extruded into plates with cross-sectional dimensions of 12 mm × 48 mm (Hefei YEKEDUANYA JIXIE Corp., Hefei, China). The extruded alloy plates underwent solution treatment at 470 °C for 2 h (Tianjin Zhonghua Furnace Corp., China), followed by water quenching. The final aging stage involved T74 treatment, which consisted of aging the samples for 8 h at 120 °C, followed by re-aging for 24 h at 160 °C (Tianjin Zhonghua Furnace Corp., China).

The Vickers hardness of the alloys was evaluated using an HVS-50 tester (Changsha Huayin Testing Instruments Co., Ltd., Changsha, China), with a 5 kgf load applied for 10 s. A minimum of five measurements were recorded for each sample to determine the hardness value. Tensile properties were assessed using an Instron 3369 universal testing machine (Instron, Norwood, MA, USA) equipped with a contact extensometer at a displacement rate of 2 mm/min. The tensile specimens, measuring 80 mm × 6 mm × 2 mm, were extracted in alignment with the extrusion direction of the plate. For each testing condition, three tensile samples were analyzed. The intergranular corrosion (IGC) test was performed by immersing the specimens in a solution containing 57 g/L NaCl and 10 mL/L H_2_O_2_ at 35 °C for 6 h. EXCO tests were conducted in a solution comprising 234 g/L NaCl, 50 g/L KNO_3_, and 6.3 mL/L HNO_3_ for 48 h at 25 °C. Optical microscopy was used to characterize the corrosion damage observed in the cross-sections of the samples, which were oriented perpendicular to the extrusion direction. Additionally, electrical conductivity measurements were carried out using a 7A051 eddy current conductivity meter (Xiamen Fusite Electronic Technology Co., Ltd., Xiamen, China).

The alloys underwent physical phase analysis using the X-ray diffractometer (XRD, D8 Advance, Bruker, Bremen, Germany) with a scanning speed of 5 °/min and a scanning range of 10–90°. Scanning electron microscopy (SEM) was employed to examine the fracture surfaces of the specimens, utilizing an MIRA4 LMH model from TESCAN located in the Brno, Czech Republic. The NordlysMax3 detector, manufactured by Oxford Instruments in the Oxford, UK, was to acquire electron backscatter diffraction (EBSD) data with a step size of 1 μm. The EBSD specimens underwent electro-polishing in a mixed solution comprising 10% HClO_4_ and 90% C_2_H_5_OH (vol.%) at a voltage of 20 V to eliminate the mechanically polished layer. Transmission electron microscopy (TEM) analyses were conducted using a Talos F200X apparatus from Thermo Fisher Scientific, Waltham, MA, USA, which was equipped with a Super-X energy-dispersive X-ray spectroscopy (EDS) system, operating at 200 kV. For TEM sample preparation, the specimens were mechanically ground to produce 70 μm thin foils, which were subsequently punched into 3 mm discs. These discs were then subjected to twinjet electro-polishing in a solution consisting of 30% HNO_3_ and 70% CH_3_OH (vol.%) at a temperature of −30 °C and a voltage of 20 V.

## 3. Results and Discussion

### 3.1. As-Extruded Microstructure

**Figure 1:**
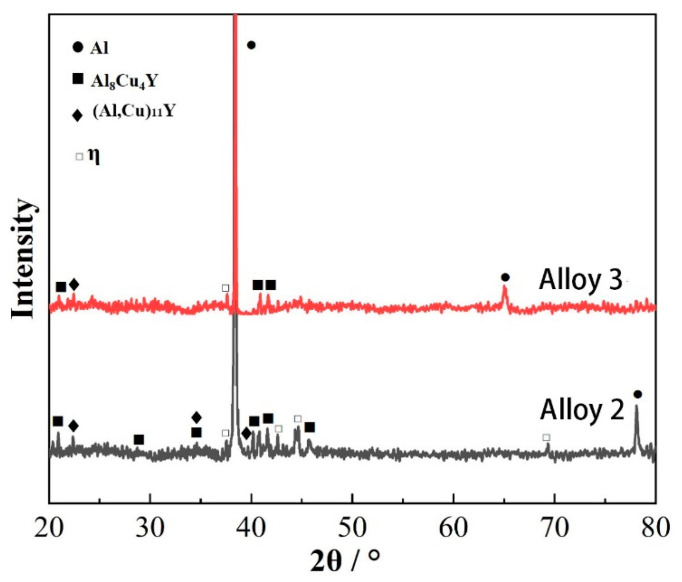
XRD graph of as-extruded alloys 2 and 3.

Previous research indicates that the primary phases in the extruded 7Al-5.6Zn-2.5Mg-1.6Cu-0.20Cr aluminum alloy are Al and MgZn_2_ phases [12,13]. After adding 0.1% Y, XRD patterns of Alloy 2 indicate that the main phases in the alloy are Al and MgZn_2_, accompanied by small amounts of Al_8_Cu_4_Y and (Al,Cu)_11_Y_3_ (Figure 1). Increasing the Y content further to 0.2% results in Alloy 3 being composed of Al, MgZn_2_, and Al_8_CuY. Thermodynamic phase diagram calculations for the Al-Cu-Y ternary system by Zhang et al. [7] suggest the possible formation of Al_8_Cu_4_Y and (Al,Cu)_11_Y_3_ in these alloys.

**Figure 2:**
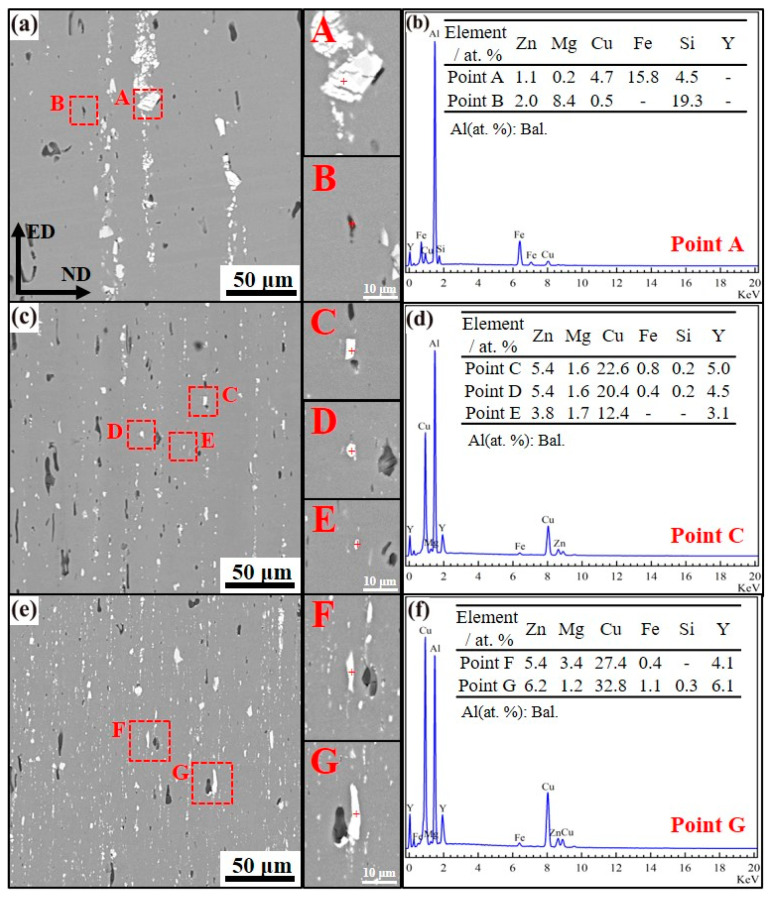
SEM images and EDS analysis of the extruded alloys 1 (**a**,**b**), 2 (**c**,**d**), and 3 (**e**,**f**).

**Figure 3:**
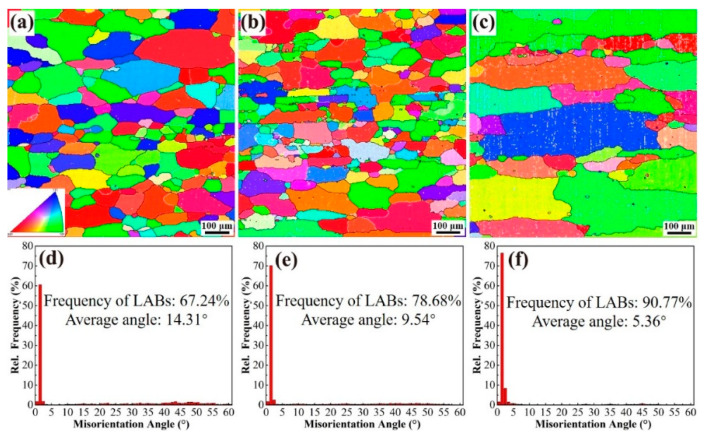
IPF diagrams and grain boundary orientation difference statistical diagrams of alloys 1 (**a**,**d**), 2 (**b**,**e**), and 3 (**c**,**f**) after the solution treatment.

Figure 2 presents scanning electron microscopy (SEM) micrographs coupled with energy dispersive spectroscopy (EDS) mapping for extruded alloys designated as 1, 2, and 3. Metallographic characterization demonstrates pronounced textural anisotropy in all specimens, manifested by elongated second-phase particles exhibiting textured alignment parallel to the extrusion axis. Comparative analysis reveals significant morphological differentiation: Alloy 1 (Figure 2a) contains coarse intermetallic aggregates with spatial clustering tendencies, whereas Alloys 2 (Figure 2c) and 3 (Figure 2e) display refined secondary phases demonstrating homogeneous dispersion characteristics. Phase compositional profiling through EDS quantification identifies two distinct intermetallic populations in Alloy 1: (i) Al_7_Cu_2_Fe intermetallics exhibiting bright contrast and (ii) Mg_2_Si precipitates with dark imaging response. The Y-modified Alloys 2 and 3 exhibit additional rare-earth-containing phases, where phase dimensions demonstrate a positive correlation with Y concentration—increasing from 0.4 μm (Point E) to 1.2 μm (Points C/G). Thermomechanical processing induces significant phase morphology evolution: High-Y phases develop lamellar structures through severe plastic deformation, while low-Y variants retain equiaxed configurations. Mechanistically, dynamic fragmentation during hot extrusion generates submicron precipitates (0.4–0.8 μm) that effectively promote grain refinement via particle-stimulated nucleation (PSN). This phenomenon occurs through localized strain accumulation at particle-matrix interfaces, facilitating dynamic recrystallization through strain-induced boundary migration mechanisms [7,8]. The addition of Y leads to a reduction in the size and a more dispersed distribution of the intermetallic compounds Al_7_Cu_2_Fe and Mg_2_Si in the extruded 7075 alloy, which contributes to the improvement of the alloy’s corrosion resistance.

### 3.2. Microstructure After Solution Treatment

Figure 3 systematically illustrates the inverse pole figure (IPF) mappings and corresponding statistical distributions of grain boundary misorientation angles for the solution-treated alloys 1, 2, and 3. Microstructural characterization reveals that all specimens maintain residual grain elongation along the extrusion direction, a characteristic feature inherited from the severe plastic deformation during hot extrusion, manifested by substantially elevated aspect ratios. Notably, alloys 1 (Figure 3a) and 2 (Figure 3b) exhibit bimodal grain size distributions comprising both coarse deformed grains and recrystallized fine equiaxed grains after thermo-mechanical processing. In marked contrast, alloy 3 (Figure 3c) displays significant abnormal grain growth phenomena, where the microstructure is dominated by exceptionally large grains with limited residual fine grains sporadically distributed at triple junctions. Quantitative analysis of grain boundary characteristics demonstrates progressive enhancement of low-angle grain boundary (LAGB, 2°–15°) fractions across the alloy series. Specifically, the LAGB proportions measure 67.24, 78.68, and 90.77 for alloys 1, 2, and 3, respectively, indicating a strong correlation between alloy composition and dislocation recovery behavior during solution treatment. This systematic variation in boundary misorientation distribution suggests differential recrystallization kinetics and strain energy storage capabilities among the investigated alloy systems.

Furthermore, the average grain orientation difference exhibits a decreasing trend, with values of 14.31° for alloy 1, decreasing to 9.54° for alloy 2, and reaching a minimum of 5.36° for alloy 3. Additionally, the predominant fiber texture orientation for alloy 1 post-extrusion is aligned with the <101> direction. With the introduction of a small amount of Y, the fiber orientation in alloy 2 shifts, showing a decrease in the <111> direction and an increase in the <001> direction. With further increases in Y content, alloy 3 predominantly exhibits fiber orientation in the <101> direction, accompanied by a slight increase in the <111> direction and a decrease in the <001> direction.

**Figure 4:**
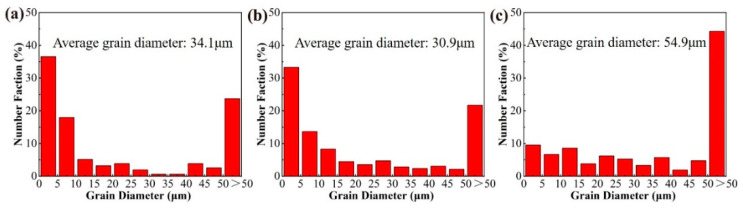
The Grain size distribution diagrams of the alloys 1 (**a**), 2 (**b**), and 3 (**c**).

Upon completion of the solution treatment, the grain size distributions of the investigated alloys are depicted in Figure 4. In alignment with the microstructural observations presented in Figure 3, alloys 1 (Figure 4a) and 2 (Figure 4b) demonstrate a predominant population of fine grains, with 40% to 50% of the grains having diameters below 10 µm. Furthermore, approximately 20% of the grains in these alloys exhibit diameters exceeding 50 µm, resulting in mean grain sizes of 34.1 µm and 30.9 µm, respectively. In stark contrast, alloy 3 (Figure 4c) manifests substantial grain coarsening, evidenced by a marked increase in the proportion of grains larger than 50 µm to 45%, concomitant with a reduction in the fraction of fine grains, culminating in an average grain size of 54.9 µm. These findings suggest that the addition of a limited quantity of yttrium (Y) facilitates the formation of fine, equiaxed grains, thereby refining the overall grain structure. However, excessive Y content induces accelerated grain growth and coarsening. This phenomenon may be attributed to the excessive accumulation of Y within the aluminum matrix, which could lead to the formation of detrimental secondary phases or disrupt the spatial distribution and synergistic interactions of other alloying elements [8,10], ultimately impeding the grain refinement process. Therefore, precise control of Y content in aluminum alloys is imperative to achieve optimal grain refinement and enhance the resultant mechanical properties.

**Figure 5:**
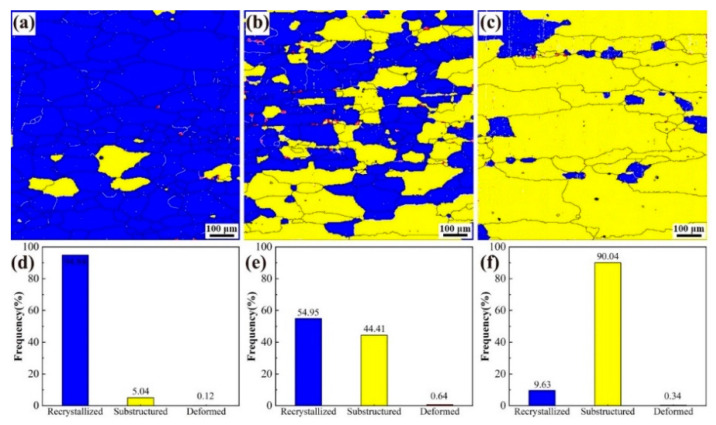
Statistical chart of recrystallization structure distribution and proportion of the alloys 1 (**a**,**d**), 2 (**b**,**e**), and 3 (**c**,**f**) after solution treatment.

Figure 5 delineates the recrystallization microstructure evolution in the alloys, with quantitative phase distribution analyses of recrystallized (RXed), substructured, and deformed regions. Alloy 1 (Figure 5a,d) manifests predominant RXed grains (94.8 vol%) with limited substructures (5.0 vol%), demonstrating near-complete recrystallization. In comparison, Alloy 2 (Figure 5b,e) reveals a dual-phase configuration comprising 54.9 vol% RXed and 44.4 vol% substructured grains. Notably, Alloy 3 (Figure 5c,f) exhibits phase inversion with substructured domains occupying 90.0 vol%, while RXed regions diminish to 9.6 vol%. The deformation content remains below 1 vol% across all alloys, peaking at 0.64 vol% in Alloy 2. This microstructural transition implies that Y addition effectively suppresses reverse recrystallization through the Zener pinning mechanism, as evidenced by accelerated RXed-to-substructure conversion kinetics (Alloy 3: 90.4% transition efficiency vs. Alloy 1: 5.0%), aligning with the Y segregation effects reported by Mochugovskiy et al. [14].

**Figure 6:**
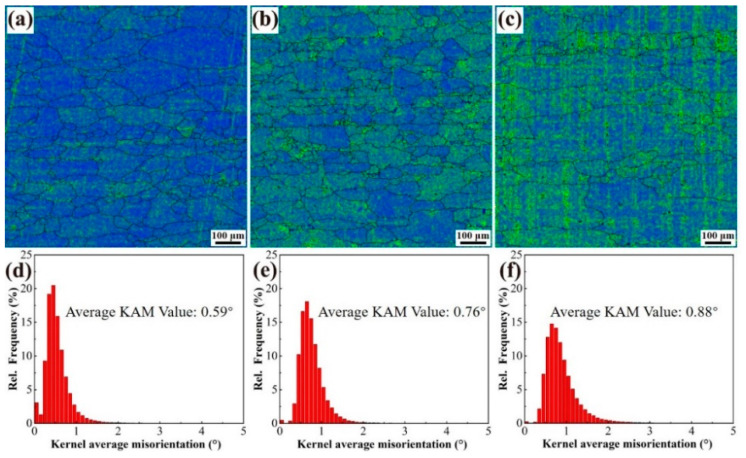
KAM images and statistical diagrams of the alloys 1 (**a**,**d**), 2 (**b**,**e**), and 3 (**c**,**f**).

Figure 6 presents the kernel average misorientation (KAM) diagrams along with the statistical distribution of the local mismatch degree within the internal structure of the alloys. The KAM values of the three alloys are 0.59°, 0.76°, and 0.88°, respectively. During the high-temperature process, the internal slip occurs within the alloy, leading to the entanglement of numerous dislocations, which subsequently form a cellular structure and contribute to the development of the subcrystalline architecture [15]. The KAM analysis serves to illustrate the geometric necessity of dislocations (GND) induced by thermal deformation in the alloys, and the GND density for each alloy sample can be calculated accordingly [16].

$$\rho_{GND}=\frac{2<\theta >}{bd}$$

where *b* is the Platonic vector, which is calculated to be 2.8637 nm for Al, and *d* is the scanning step during the sample testing, all of which are 1.5 µm. *θ* is a constant, which is the radian value of the KAM depending on the type of dislocations.

Substituting the data into the formula and calculating gives the GND densities of the alloys 1, 2, and 3 as 2.75 × 10^14^ m^−2^, 3.54 × 10^14^ m^−2^, and 4.10 × 10^14^ m^−2^, respectively. Therefore, the addition of the Y element will result in a significant increase in the GND density of the alloys.

### 3.3. Age Precipitation

**Figure 7:**
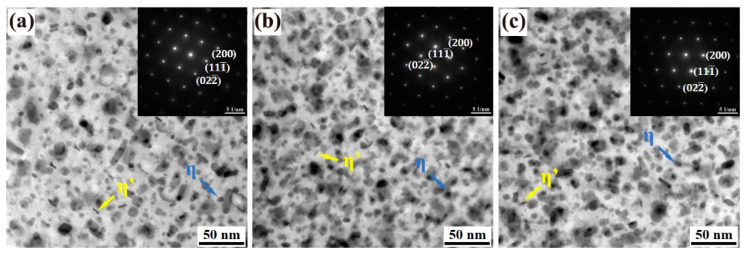
TEM images and selected area electron diffraction spot pattern of the alloys 1 (**a**), 2 (**b**), and 3 (**c**) after T74 aging treatment at [110]_Al_ diffraction direction.

**Figure 8:**
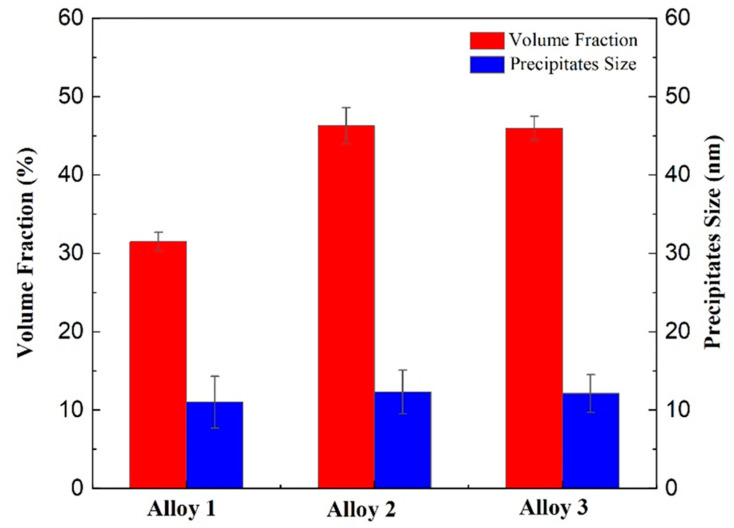
Statistical diagram of intragranular precipitation phases after T74 aging treatment of the alloys 1, 2, and 3.

Figure 7 displays TEM images and corresponding SAED patterns of the grain interiors for Alloys 1, 2, and 3 following the T74 aging treatment, with the grain band axis oriented along the [110]_Al_ direction. The analysis confirms the presence of intragranular precipitation in three alloys. The SAED patterns reveal that the precipitated phases predominantly consist of the metastable nanoscale η′ (MgZn_2_) phase and the larger equilibrium η (MgZn_2_) phase, accompanied by a distribution of coarser particles. Notably, the η′ phase maintains coherence with the aluminum matrix, while the η phase is non-coherent with the matrix [2]. A comparative assessment of the intragranular precipitation highlights that Alloys 2 (Figure 7b) and 3 (Figure 7c) exhibit a significantly higher density of precipitates compared to Alloy 1 (Figure 7a). Quantitative analysis of the number and size of the precipitated phases was performed using Image J, and the results are summarized in Figure 8. Histogram analysis further underscores that the differences in the number and size of precipitates between Alloys 2 and 3 are minimal, particularly when contrasted with the substantially lower density of precipitated phases observed in Alloy 1. These findings suggest that the incorporation of yttrium (Y) significantly enhances the aging precipitation characteristics of the alloys, resulting in a greater abundance of internal strengthening phases after the aging process. This enhancement is attributed to the role of Y in promoting precipitate formation, thereby contributing to improved mechanical properties in the alloys.

**Figure 9:**
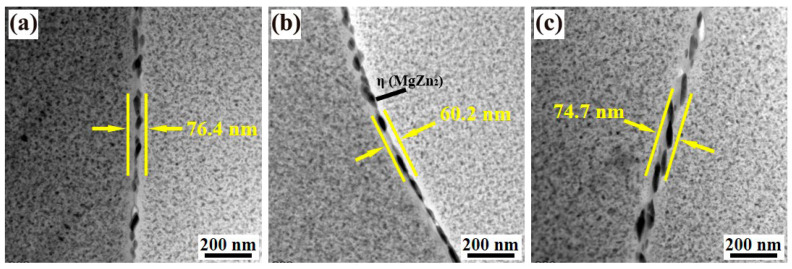
TEM bright field images at grain boundaries of the alloys 1 (**a**), 2 (**b**), and 3 (**c**) after T74 aging treatment.

Figure 9 presents TEM images of the grain boundaries in Alloys 1, 2, and 3 following T74 aging treatment. The analysis reveals distinct variations in the characteristics of the precipitate-free zone (PFZ) and grain boundary precipitation (GBP). In Alloy 1 (Figure 9a), the PFZ width measures 76.4 nm, with the GBP consisting of discontinuous equilibrium η (MgZn_2_) phases exhibiting considerable inter-precipitate spacing. In contrast, the introduction of yttrium (Y) in Alloy 2 (Figure 9b) leads to a significant reduction in the PFZ width to 60.2 nm, accompanied by a slight decrease in the spacing of GBP phases. Further increasing the Y content in Alloy 3 (Figure 9c) results in a PFZ width of 74.7 nm, alongside a notable enlargement of the GBP phases and a tendency toward continuous precipitation along the grain boundaries. These microstructural changes are closely associated with subsequent variations in tensile and corrosion properties. The findings suggest that the addition of a minor quantity of Y effectively reduces the PFZ width in the aged alloy, thereby influencing its mechanical and corrosion behavior. This effect is likely attributed to the role of Y in modifying the precipitation kinetics and distribution of GBP phases, highlighting its potential in tailoring the material properties of aluminum alloys.

### 3.4. Tensile Properties

**Figure 10:**
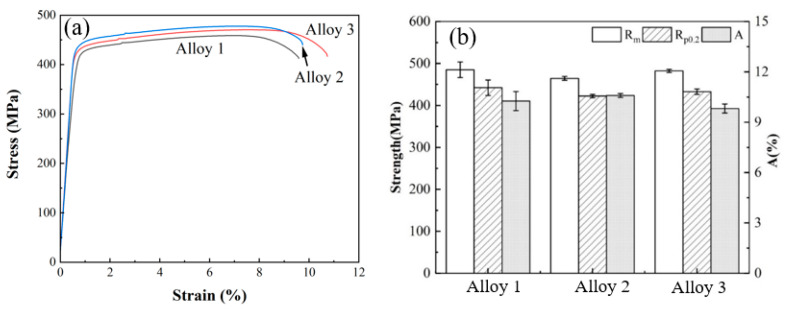
The engineering stress-strain curves (**a**) and tensile properties (**b**) of alloys 1, 2, and 3 after T74 aging treatment.

**Figure 11:**
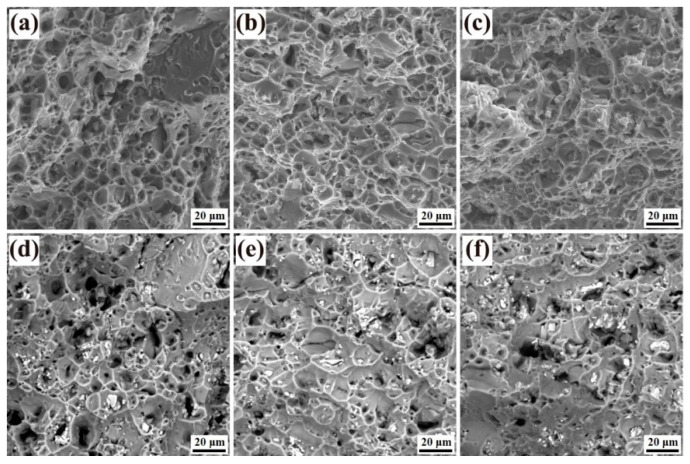
Tensile fracture morphology of alloys 1 (**a**,**d**), 2 (**b**,**e**), and 3 (**c**,**f**) after T74 aging treatment.

Figure 10 presents the tensile stress-strain curves and corresponding tensile properties of Alloys 1, 2, and 3 following T74 aging treatment. The tensile properties of all three alloys demonstrated significant improvement post-aging compared to their pre-treatment states. However, the incorporation of yttrium (Y) had a limited influence on the tensile strength, yield strength, and elongation of the aged alloys, exhibiting only marginal variations. The tensile strength of Alloys 1 to 3 was approximately 500 MPa, initially showing a slight decline before increasing slightly, with differences among the alloys remaining within a narrow range of ~15 MPa. A similar trend was observed in the yield strength, which stabilized between 430 and 440 MPa. Alloy 2 exhibited a relatively higher elongation of 10.6%, while the other two alloys achieved elongation values close to 10%. The fracture morphology of the alloys, as depicted in Figure 11, includes secondary electron images of the fracture surfaces (above) and corresponding backscattered electron images (below). The analysis reveals a significant presence of dimples (tough nests) across the fracture surfaces of all three alloys. The larger and deeper dimples, characterized by the absence of cleavage facets and shear bands, indicate a predominantly ductile fracture mode, which contributes to the enhanced elongation and plasticity of the alloys. Notably, Alloys 1 (Figure 11a,d) and 3 (Figure 11c,f) exhibit smaller dimples, whereas Alloy 2 (Figure 11b,e) features larger dimples, correlating with its superior elongation compared to the other two alloys. The backscattered electron images further reveal the presence of second-phase particles within the dimples, which are reduced in size and exhibit a tendency toward a more diffuse distribution. These findings highlight the role of microstructural features, such as dimple size and second-phase particle distribution, in influencing the tensile and fracture behavior of the alloys. The minimal impact of Y on tensile properties underscores its potential to enhance other performance characteristics, such as corrosion resistance or grain refinement, without significantly altering mechanical strength.

Tensile properties indicate that the addition of the rare earth element Y has a minimal impact on the strength of Al-5.6Zn-2.5Mg-1.6Cu-0.2Cr alloy but significantly improves its elongation. This enhancement is attributed to the ability of rare earth elements to refine the grain structure and reduce the size of brittle phases such as Al_7_Cu_2_Fe and Mg_2_Si, promoting their dispersion and thereby significantly improving the plasticity of the aluminum alloy.

### 3.5. Corrosion Behavior

**Figure 12:**
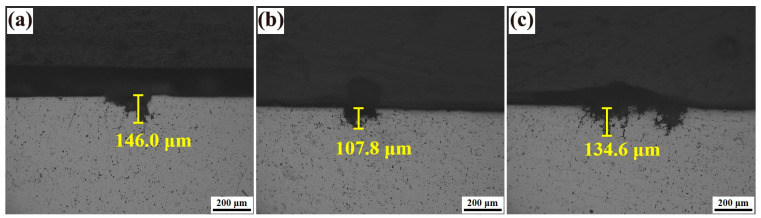
Images of intergranular corrosion morphology of the alloys 1 (**a**), 2 (**b**), and 3 (**c**) after T74 aging treatment.

Figure 12 displays the maximum depth of intergranular corrosion observed in metallographic cross-sections of Alloys 1, 2, and 3 following T74 treatment. The corrosion depth for Alloy 1 (Figure 12a) measures 146.0 μm, exhibiting extensive pitting with corrosion pathways propagating along coarse second-phase particles into the grain structure. In contrast, the incorporation of yttrium (Y) into the base 7075 alloys significantly enhanced the intergranular corrosion resistance of Alloys 2 (Figure 12b) and 3 (Figure 12c), reducing the corrosion depths to 107.8 μm and 134.6 μm, respectively. Alloy 2, in particular, exhibited a less pronounced reticulated corrosion morphology, the lowest corrosion depth, and superior corrosion resistance among the three alloys. These results demonstrate that the addition of a minor quantity of Y effectively improves the intergranular corrosion characteristics of the alloy. However, excessive Y content can lead to a reduction in intergranular corrosion resistance. A suitable amount of the rare earth element Y refines the grain structure of aluminum alloys, enhancing their resistance to intergranular corrosion. Nevertheless, when the Y content exceeds a critical threshold, its grain refinement effect and corrosion resistance benefits may saturate or diminish. Excessive Y can promote the formation of coarse rare-earth compound phases, which disrupt the distribution and behavior of other alloying elements such as Zn, Mg, and Cu, ultimately diminishing intergranular corrosion resistance [12]. In summary, optimizing the Y content in aluminum alloys is crucial to achieving enhanced microstructural refinement and superior corrosion resistance, particularly against intergranular corrosion, without compromising material performance.

**Figure 13:**
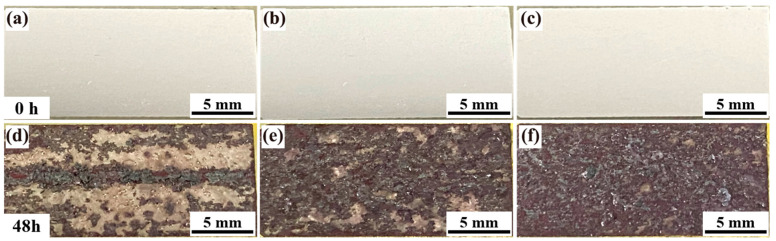
Macrophotographs of the alloys 1 (**a**,**d**), 2 (**b**,**e**), and 3 (**c**,**f**) before (**a**,**b**,**d**) and after exfoliation corrosion (**d**,**e**,**f**).

Figure 13 presents macrophotographs of Alloys 1, 2, and 3 before and after exfoliation corrosion testing, revealing a clear progression in the severity of surface exfoliation corrosion from Alloy 1 to Alloy 3. Alloy 1 (Figure 13a,d) retains a substantial amount of metallic luster across its surface despite localized pitting and skin bursting, with relatively shallow corrosion penetration. This behavior corresponds to a PB classification for exfoliation corrosion. In contrast, Alloy 2 (Figure 13b,e) demonstrates a significant reduction in resistance to exfoliation corrosion. The surface exhibits extensive scarring and minimal retention of metallic luster, with corrosion penetrating deeply into the metal, resulting in a PC classification. Alloy 3 (Figure 13c,f) displays the most severe exfoliation corrosion, with the surface entirely covered by corrosion products and a complete loss of metallic luster, leading to an EA classification. These observations indicate that the incorporation of yttrium (Y) into the alloy detrimentally affects its resistance to exfoliation corrosion. The progressive increase in corrosion severity from Alloy 1 to Alloy 3 suggests that higher Y content exacerbates the susceptibility of the alloy to exfoliation corrosion. This phenomenon may be attributed to the formation of coarse second-phase particles or altered microstructural features induced by Y, which facilitate the initiation and propagation of corrosion pathways. In summary, while Y can enhance certain properties of aluminum alloys, its addition appears to compromise exfoliation corrosion resistance, highlighting the need for careful optimization of Y content to balance performance characteristics.

The above results indicate that the addition of minor amounts of rare earth Y elements affects the microstructure, mechanical properties, and corrosion behavior of Al-5.6Zn-2.5Mg-1.6Cu-0.2Cr aluminum alloy in the extruded state. The reasons are as follows:

(1) Beyond their function in purifying the melt, rare earth Y element significantly influences the microstructure, aging behavior, recrystallization kinetics, and mechanical properties of Al-Zn-Mg-Cu series aluminum alloys. Specifically, the incorporation of yttrium (Y) increases the presence of secondary phases within the alloy, leading to the formation of finely dispersed secondary phases. These dispersed phases act as pinning agents at grain boundaries, inhibiting boundary migration and promoting grain refinement. Additionally, yttrium can interact with copper (Cu) to form intermetallic phases such as Al_8_Cu_4_Y and (Al, Cu)_11_Y [17]. During hot extrusion deformation, these phases are fragmented into particles with dimensions ranging from 0.4 to 0.8 μm. These particles further enhance grain refinement through the particle-stimulated nucleation effect, which facilitates the formation of new grains by providing nucleation sites during recrystallization. These mechanisms collectively contribute to the microstructural refinement and enhanced mechanical properties of Al-Zn-Mg-Cu alloys containing yttrium. The ability of Y to modify phase formation and grain structure underscores its potential as an effective alloying element for optimizing the performance of high-strength aluminum alloys.

(2) The atomic radius of the rare earth element yttrium (Y) is 0.18 nm, significantly larger than that of aluminum (Al) at approximately 0.14 nm. This size disparity can induce lattice distortion when Y is incorporated into the aluminum matrix, leading to solid solution strengthening [7,18]. Additionally, the formation of Y-containing dispersed phases at grain boundaries contributes to second-phase strengthening and promotes microstructural refinement by pinning grain boundaries and inhibiting their migration [18]. Despite these potential benefits, the mechanical properties of the alloy in this study did not exhibit significant improvement with the addition of Y, even though the density of precipitated phases increased markedly during aging. The fracture morphology, as illustrated in Figure 12, reveals the presence of second-phase particles within the dimples of the alloy. However, these particles are notably smaller and more uniformly dispersed compared to previous observations. This refined distribution suggests that while the incorporation of Y does not substantially enhance the strength of the alloy, it may improve its plasticity to some extent. In summary, the addition of Y primarily influences the alloy’s microstructure and phase distribution rather than its overall strength, highlighting its potential role in tailoring plasticity and microstructural characteristics rather than mechanical strength alone.

(3) Improving the corrosion resistance of aluminum alloys involves strategies to reduce the formation of GBPs by increasing the proportion of low-angle grain boundaries [19,20]. High-angle grain boundaries, characteristic of recrystallized structures, exhibit elevated interfacial energy, which facilitates the nucleation of precipitated phases. This process often results in the formation of continuous coarse GBPs and the enlargement of PFZ, both of which can degrade corrosion resistance. In contrast, subcrystalline boundaries possess lower energy, leading to reduced accumulation of precipitated phases and a narrower PFZ. These characteristics diminish the potential difference between the grain boundary and the matrix, thereby enhancing the alloy’s corrosion resistance [21]. The incorporation of yttrium (Y) in Alloys 2 and 3 increases the proportion of low-angle grain boundaries and effectively inhibits recrystallization. This promotes a faster transformation of the alloy’s internal structure to a subcrystalline form, resulting in more subcrystalline structures and improved corrosion resistance. However, excessive Y content can reduce intergranular corrosion resistance, as it increases the number of second-phase particles and their diffuse distribution within the alloy. These particles create a potential difference between the intermetallic phase and the aluminum matrix, significantly increasing corrosion susceptibility [22]. As evidenced in Figure 13, the addition of Y can also negatively impact the alloy’s exfoliation corrosion resistance. While Y enhances the overall grain structure and reduces the formation of continuous GBPs, excessive Y content introduces new challenges by promoting the formation of second-phase particles that exacerbate corrosion. Thus, optimizing Y content is critical to balance the beneficial effects of grain boundary modification and the adverse effects of second-phase particle formation on corrosion resistance.

(4) The observed improvements in the corrosion behavior of aluminum alloys due to the addition of rare earth Y elements, particularly their ability to refine grain structure and reduce brittle phase sizes, would have significant implications for real-world applications. Industries that prioritize corrosion resistance, such as aerospace, automotive, and marine engineering, would benefit from alloys with enhanced resistance to pitting, crevice, and stress-corrosion cracking.

## 4. Conclusions

(1) The addition of 0.1 wt.% yttrium to Al-5.6Zn-2.5Mg-1.6Cu-0.2Cr alloy results in a significant refinement of grain size following solid solution treatment. However, further increases in yttrium content led to notable grain coarsening. Concurrently, the proportion of low-angle grain boundaries, dislocation density, and the fraction of subgrain structures increase with higher yttrium content. The introduction of yttrium significantly enhances the aging precipitation kinetics, promoting a higher volume fraction of the η′ phase. Notably, at the yttrium content of 0.1 wt.%, the width of the precipitate-free zone is markedly reduced.

(2) Following the T74 aging treatment, the strength of yttrium-containing alloys remains stable, with all specimens exhibiting tensile strengths exceeding 450 MPa. Moreover, the elongation and plastic toughness of the alloys are improved. Specifically, the addition of 0.1 wt.% yttrium reduces the maximum depth of intergranular corrosion in the aged alloy to 107.8 μm. However, the resistance to exfoliation corrosion deteriorates with increasing yttrium content.

## Data Availability

The raw data supporting the conclusions of this article will be made available by the authors on request.
